# Africa’s challenged ENT services: highlighting challenges in Zambia

**DOI:** 10.1186/s12913-019-4267-y

**Published:** 2019-07-02

**Authors:** Lufunda Lukama, Chester Kalinda, Colleen Aldous

**Affiliations:** 1 College of Health Sciences, Nelson R. Mandela School of Clinical Medicine, Discipline of Otorhinolaryngology, University of KwaZulu-Natal, Durban, 4001 South Africa; 2 University of Namibia, Katima Mulilo Campus, Private Bag, Katima Mulilo, 1096 Namibia; 3 College of Health Sciences, Howard College Campus, School of Nursing and Public Health, University of KwaZulu-Natal, Durban, 4001 South Africa; 4 College of Health Sciences, Nelson R. Mandela School of Clinical Medicine, University of KwaZulu-Natal, Durban, 4001 South Africa

**Keywords:** Ear, Nose and throat (ENT), Equipment, Human resource, Infrastructure, Service delivery

## Abstract

**Background:**

Diseases of the ear, nose and throat (ENT) are common and are a major cause of morbidity and mortality. In many low income countries like Zambia, the high ENT disease burden has not received the required resources for treatment. We investigated ENT service provision in hospitals in Zambia by documenting the profile of hospitals offering ENT services and examining the country’s ENT services with regards to human resource, infrastructure and availability of equipment based on the levels of care of various hospitals**.**

**Methods:**

The study was a cross-sectional descriptive survey conducted using a structured and piloted questionnaire which was administered to the 109 Ministry of Health (MoH) registered hospitals across the country. Ethical clearance was granted by University of KwaZulu-Natal and the Zambia National Health Research Authority. Participation in the study was voluntary and all respondents signed informed consent. Descriptive statistics were used to analyse the data.

**Results:**

Of the 109 hospitals approached to participate in the study, 61 (55.9%) hospitals responded. This represented 83.3% (*n* = 5) of Third Level Hospitals (TLH), 89.5% (*n* = 17) of Second Level Hospitals (SLH) and 41.7% (*n* = 35) of First Level Hospitals (FLH) countrywide. Of the participating hospitals, 6.6% (*n* = 4) were unclassified. Within this sample, 8.6% (*n* = 3) FLH, 11.8% (*n* = 2) SLH and 60.0% (*n* = 3) TLH had an ENT examination room. Only 2.9% (*n* = 4) hospitals had an audiology booth and 1.6% (*n* = 1) had a speech therapy room. Of the second and third level hospitals, 9.1% (*n* = 2) had flexible rhinolaryngoscopes, 18.2% (*n* = 4) had operating microscopes and 68.2% (*n* = 15) adenotonsillectomy sets. The data revealed that there were 4 ENT surgeons, 1 Audiologist and no Speech Therapists across the country.

**Conclusion:**

Zambia’s ENT services were deficient at all levels of hospital care. There were deficiencies in infrastructure, human resource and equipment in hospitals. With the current burden of disease, critical intervention is required. These findings should be used to direct national policy on the improvement of ENT service provision in Zambia.

**Electronic supplementary material:**

The online version of this article (10.1186/s12913-019-4267-y) contains supplementary material, which is available to authorized users.

## Background

Diseases of the ear, nose and throat (ENT) are universally a major public health concern [1–3]. The Global Burden of Disease (GBD), including ENT conditions, has uniformly been reported to be proportional to resource deprivation [4, 5]. Even though developed countries are not exempt from the high ENT disease burden [6], low income African and Asian countries are most affected [7–9]. Globally, disabling hearing loss affects about 466 million people [10], debilitating individual sufferers, families and countries alike [9]. Head and neck cancer, the 9th most common malignancy, has high mortality rates in developing countries [11]. In the majority of places, individuals with communication difficulties are not prioritized in health care systems as governments focus on saving lives as opposed to improving quality of life [12].

The high ENT disease burden has not been adequately addressed, with ENT conditions not specifically coded for within the framework governing GBD estimates [4, 5]. Zambia, a country with high ENT disease burden and HIV prevalence, has only recently listed ENT conditions as priority areas in its latest National Health Strategic Plan (NHSP) [13]. Despite 11.5% of school children in part of the nation’s capital having impaired hearing [14], emerging evidence suggests the unavailability of an essential drug list for ENT [15]. With much of the national health resources directed towards infectious and non-communicable diseases, the inertia in addressing ENT diseases has led to significant preventable morbidity and mortality across the country [13].

The challenges facing delivery of ENT services in low income counties like Zambia include meager health budgets, poor infrastructure and equipment and shortage of trained medical personnel [4, 16–18]. In Zambia, ENT is amongst the least resourced surgical disciplines for which the government has prioritised to address in its latest NHSP [13]. Despite previous studies having highlighted deficits in the Zambian ENT service [5, 18, 19], there has as yet not been a comprehensive audit to describe the current state of the country’s existing ENT resources at all levels of health care, including primary, secondary and tertiary hospitals. Thus, the challenges facing the ENT service in Zambia have likely been underestimated. This study determined the current status of ENT service provision in hospitals in Zambia by documenting the profile of hospitals offering ENT services and examining the ENT service with regards to human resource, infrastructure and availability of equipment based on the levels of care of the various hospitals. It is hoped that this data will provide impetus to direct national policy on the improvement of ENT.

## Methods

A cross-sectional descriptive survey was conducted using a structured questionnaire (Additional file 1) that was derived from previous studies [5, 20, 21], trialed in the Durban metropolitan area and validated by 5 ENT consultants within the Province of KwaZulu-Natal in South Africa. It was then modified to be appropriate to the Zambian setting. Medical Officer-In-Charges (MOIC) or the most appropriate health workers with knowledge of the ENT service of individual hospitals in Zambia registered with the country’s Health Ministry performed a physical count of the infrastructure and equipment relevant to the study and completed the questionnaire. The infrastructure and equipment surveyed included that necessary for provision of ENT services at first, second and third level hospital care. Where necessary, images of equipment were confirmed with the authors by email and video conference calls. Information regarding human resource was provided by the hospital’s Human Resource Officers (HROs) and validated with data obtained from the Health Professions Council of Zambia (HPCZ). To ensure response accuracy and uniformity, study participants referred to the description of terms outlined in the questionnaire. (Additional file 2). The survey was conducted between 17th January 2017 and 2nd January 2018.

The questionnaire was distributed to the hospitals via the MOIC’s offices using either email, post or hand delivery. Contact details of all MOIC or the most appropriate health workers who were the study participants, were retrieved from the country’s Health Ministry headquarters. Telephonic and email reminders were sent to the participants a minimum of three times until all answered questionnaires were returned. Participation in the study was voluntary and all respondents signed informed consent. Ethical approval was obtained from the University of KwaZulu-Natal Research Ethics Committee and the Zambia National Health Research Authority. Collected data was organized and analyzed using SPSS version 25 (IBM Corp. Released 2016. IBM SPSS Statistics for Macintosh, Version 25.0. Armonk, NY: IBM Corp.).

Descriptive statistical analysis was carried out focusing on the profile of the hospitals that were sampled. The study further determined the availability of infrastructure and that of essential ENT equipment.

The manuscript was written using the STROBE checklist for cross sectional studies [22]. (Additional file 3).

## Results

### Profile of hospitals

A total of 61 of the 109 hospitals in Zambia participated in the study. This represented 83.3% (*n* = 5) of TLH, 89.5% (*n* = 17) of SLH and 41.7% (*n* = 35) of FLH. In addition, 62.3% (*n* = 38) of the participating hospitals were public (PH), 18.0% (*n* = 11) private (PrH) and 19.7% (*n* = 12) faith based organisation (FBO) owned. However, 6.6% (*n* = 4) of the participating hospitals were not classified as FLH, SLH or TLH. The majority of participating hospitals (26.2%, *n* = 16) were from Lusaka (LSK) Province while the least (3.3%, *n* = 2) were from Muchinga (Much) and Luapula (Lua) Provinces. The rest of the participating hospitals were from Southern (South) Province (13.1%,*n* = 8), Western (West) Province (11.5%, *n* = 7), Copperbelt (CB) Province (11.5%, *n* = 7), Eastern (East) Province (9.8%, *n* = 6), North-Western (N-West) Province (8.2%, *n* = 5), Northern (North) Province (6.6%, *n* = 4) and Central (Cent) Province (6.6%, *n* = 4).

## Human resource

In Zambia there were a total of four ENT surgeons, one audiologist and no speech therapist registered with the Health Professions Council of Zambia. Table 1 outlines the distribution of ENT related human resource in hospitals in Zambia.

**Table 1:** Distribution of ENT related human resource in surveyed hospitals in Zambia

Core ENT workforce	Absolute count (n)	Count (n) registered with HPCZ^a^	Countrywide distribution and comments
Specialist (qualified) ENT surgeons	7	4^a^	7 from hospital responses – 4 in Lusaka Province, 1 in Copperbelt Province, 1 in Central Province and 1 in Western Province
Registrars stationed in ENT	0	–	
Non-specialist medical doctors not in specialist training stationed in ENT	11	–	3 in Copperbelt Province and 8 shared among 3 hospitals in Lusaka urban district
Medical Licentiates	0	–	
Clinical officers formally trained in ENT	5	–	3 based in Copperbelt Province, 1 in Eastern Province, 1 in Western Province
Audiologists	2	1^a^	
Speech therapists	1	0^a^	A Lusaka hospital reported having 1 HPCZ unregistered speech therapist
Nurses dedicated to ENT	8	–	3 in Copperbelt, 2 in Central, 2 in Lusaka and 1 in Western Provinces
Supportive Professionals			
Plastics and reconstructive surgeons	1	1^a^	
Neurosurgeons	2	2^a^	Both based in Lusaka city
Ophthalmologists	20	25^a^	
Thoracic surgeons	0	0^a^	
Maxillofacial Surgeons	3	3^a^	
Vascular surgeons	0	0^a^	
General surgeons	30	38^a^	

^a^ Registered with the HPCZ as of January, 2018

## Infrastructure

Of the participating hospitals, 6.6% (*n* = 4) had an audiology booth and speech therapy room. It was also observed that 8.6% (*n* = 3) of FLH, 11.8% (*n* = 2) SLH, 80% (*n* = 4) of TLH had operating time dedicated to ENT. Table 2 summarises the distribution of ENT relevant infrastructure among hospitals in Zambia.

**Table 2:** Infrastructure distribution according to levels of care and status of hospitals in Zambia

	FLH (*n* = 31)	SLH (*n* = 17)	TLH (*n* = 5)	Public *(n* = 38)	Private (*n* = 11)	FBO owned (*n* = 12)	Unclassified by respondents *n* = 4
ENT examination room	8.6% *n* = 3	11.8% *n* = 2	60% *n* = 3	13.2% *n* = 5	27.3% *n* = 3	8.3% *n* = 1	2.9% *n* = 1
Operating theatre	94.3% *n* = 33	100% *n* = 17	100% *n* = 5	94.7% *n* = 36	100% *n* = 11	83.3% *n* = 10	100% *n* = 4
Audiology booth	2.9% *n* = 1	5.9% *n* = 1	40% *n* = 2	5.3% *n* = 2	9.1% *n* = 1	8.3% *n* = 1	0
Speech therapy room	2.9% *n* = 1	0	0	0	0	8.3% *n* = 1	0
Intensive/critical care facility	20% *n* = 7	64.7% *n* = 11	80% *n* = 4	31.6% *n* = 12	45.5% *n* = 5	50% *n* = 5	2.9% *n* = 1
High Dependency (care) facility	17.1% *n* = 6	47.1% *n* = 8	80% *n* = 4	31.6% *n* = 12	27.3% *n* = 3	33.3% *n* = 4	2.9% *n* = 1

## Equipment

Regarding basic ENT equipment, the study showed that 73.8% (*n* = 45) of the surveyed hospitals had at least one otoscope, 16.4%(*n* = 10) at least a pneumatic bulb, 19.7%(*n* = 12) had at least a single 512 Hz tuning fork, 73.8% (*n* = 45) suction machine(s) and 59.0% (*n* = 36) at least a syringing kit (Table 3).

**Table 3:** Percentage of surveyed hospitals with at least one of the basic ENT ear equipment according to provinces

	Percentage (%) by Province										
Ear Equipment	CB	LSK	East	N-West	Much	North	Lua	South	Cent	West	Total
Otoscope(s)	85.7% *n* = 6	68.9% *n* = 11	50% *n* = 3	60% *n* = 3	100% *n* = 2	25% *n* = 1	100% *n* = 2	87.5% *n* = 7	75% *n* = 3	100% *n* = 7	73.8% *n* = 45
Pneumatic bulb(s)	28.6% *n* = 2	37.5% *n* = 6	0	20% *n* = 1	0	0	0	0	0	14.3% *n* = 1	16.4% *n* = 10
256 Hz tuning fork(s)	14.3% *n* = 2	31.3% *n* = 5	0	40% *n* = 2	0	0	0	25% *n* = 2	0	14.3% *n* = 1	18.0% *n* = 11
512 Hz tuning fork(s)	0	50% *n* = 8	0	40% *n* = 2	0	0	0	12.5% *n* = 1	25% *n* = 1	0	19.7% *n* = 12
1024 Hz tuning fork(s)	14.3% *n* = 1	6.3% *n* = 1	0	0	0	0	0	12.5% *n* = 1	0	14.3% *n* = 1	6.6% *n* = 4
Barany box (es)	0	6.3% *n* = 1	0	0	0	0	0	0	0	14.3% *n* = 1	3.3% *n* = 2
Ear syringing kit(s)	85.7% *n* = 6	75% *n* = 12	83.3% *n* = 5	40% *n* = 2	0	0	100% *n* = 2	75% *n* = 6	25% *n* = 1	28.6% *n* = 2	59.0% *n* = 36
Ear hook(s)	28.6% *n* = 2	56.3% *n* = 9	16.7% *n* = 1	0	0	0	50% *n* = 1	50% *n* = 4	25% *n* = 1	14.3% *n* = 1	31.2% *n* = 19
Jobson horne probe(s)	14.3% *n* = 2	50% *n* = 8	0	0	0	0	0	0	0	14.3% *n* = 1	16.4% *n* = 10

*Province abbreviations*: *CB* Copperbelt, *LSK* Lusaka, *East* Eastern, *N-West* North-Western, *Much* Muchinga, *North* Northern, *Lua* Luapula, *South* Southern, *Cent* Central, *West* Western

The availability of basic ENT nose and sinus equipment varied between hospitals (Table 4). Only 39.3% (*n* = 24) of hospitals stocked at least one nasal speculum while 37.7% (*n* = 23) had nose packing forceps. In addition, 29.5% (*n* = 18) of hospitals surveyed had biopsy forceps. Hospitals based in Lusaka Province were the most stocked with 62.5% (*n* = 10) having had nasal speculum(s) and nose packing forcep(s), and 43.8% (*n* = 7) having biopsy forcep(s). Hospitals based in North-Western and Muchinga Provinces had none of the basic ENT nose and sinus equipment.

**Table 4:** Percentage of surveyed hospitals with at least one of the basic ENT nose and sinus equipment according to provinces

Equipment	% by Province										
Nose and sinus equipment	CB	LSK	East	N-West	Much	North	Lua	South	Cent	West	Total
Nasal speculum(s)	28.6% *n* = 2	62.5% *n* = 10	66.7% *n* = 4	0	0	25% *n* = 1	0	50% *n* = 4	25% *n* = 1	28.6% *n* = 2	39.3% *n* = 24
Nose packing forcep(s)	42.9% *n* = 3	62.5% *n* = 10	66.7% *n* = 4	0	0	25% *n* = 1	0	50% *n* = 4	25% *n* = 1	14.3% *n* = 1	37.7% *n* = 23
Biopsy forcep(s)	28.6% *n* = 2	43.8% *n* = 7	66.7% *n* = 4	0	0	25% *n* = 1	50% *n* = 1	25% *n* = 2	0	14.3% *n* = 1	29.5% *n* = 18

*Province abbreviations*: *CB* Copperbelt, *LSK* Lusaka, *East* Eastern, *N-West* North-Western, *Much* Muchinga, *North* Northern, *Lua* Luapula, *South* Southern, *Cent* Central, *West* Western

Concerning basic ENT head and neck equipment, all surveyed hospitals in Eastern, Muchinga and Southern Provinces had suction machines while those in Luapula Province had none (Table 5). Fine needle aspiration cytology (FNAC) apparatus were scarce, with 8.2% (*n* = 5) of hospitals stocking them. Overall, 27.9% (*n* = 17) of the hospitals stocked examination lights/reflecting mirror(s), 37.7% (*n* = 23) stocked laryngeal mirror(s), 62.3% (*n* = 38) stocked tongue depressor(s) while 73.8% (*n* = 45) were equipped with suction machines (Table 5).

**Table 5:** Percentage of surveyed hospitals with at least one of the basic ENT head and neck equipment according to provinces

Equipment	% by Province										
Head and Neck Equipment	CB	LSK	East	N-West	Much	North	Lua	South	Cent	West	Total
Examination lights	42.9% *n* = 3	43.8% *n* = 7	0	20% *n* = 1	100% *n* = 2	25% *n* = 1	0	12.5% *n* = 1	25% *n* = 1	14.3% *n* = 1	27.9% *n* = 17
Laryngeal mirror	42.9% *n* = 3	68.9% *n* = 11	50% *n* = 3	0	50.0% *n* = 1	0	0	12.5% *n* = 1	25% *n* = 1	42.6% *n* = 3	37.7% *n* = 23
Tongue depressor	42.9% *n* = 3	93.8% *n* = 15	83.3% *n* = 5	20% *n* = 1	100% *n* = 2	75% *n* = 3	50% *n* = 1	37.5% *n* = 3	100% *n* = 4	14.3% *n* = 1	62.3% *n* = 38
Suction equipment	57.1% *n* = 4	81.3% *n* = 13	100% *n* = 6	40% *n* = 2	100% *n* = 2	75% *n* = 3	0	100% *n* = 8	50% *n* = 2	71.4% *n* = 5	73.8% *n* = 45
FNAC apparatus	0	6.3% *n* = 1	16.7% *n* = 1	0	50% *n* = 1	0	0	12.5% *n* = 1	0	14.3% *n* = 1	8.2% *n* = 5
Number Hospitals surveyed	7	16	6	5	2	4	2	8	4	7	100% *N* = 61

*Province abbreviations*: *CB* Copperbelt, *LSK* Lusaka, *East* Eastern, *N-West* North-Western, *Much* Muchinga, *North* Northern, *Lua* Luapula, *South* Southern, *Cent* Central, *West* Western

With regards to specialised ENT, audiology and speech therapy equipment, few hospitals (6.6%, *n* = 4) possessed operating microscopes, 6.6% (*n* = 4) had grommet insertion sets, 6.6% (*n* = 4) had diagnostic audiometers and 4.9% (*n* = 3) owned at least one of the following equipment: a tympanometer, screening audiometer, mastoid drill kit and mastoidectomy instrument set. Only 3.3% (*n* = 2) hospitals, both located in Lusaka Urban District, had Auditory Brain Response (ABR), Otoacoustic Emission (OAE) and Auditory Steady State Response (ASSR) equipment. Furthermore, 24.6% (*n* = 15) and 31.1% (*n* = 19) of the participating hospitals had adenotonsillectomy and tracheostomy sets, respectively. The 19 hospitals owning tracheostomy sets were spread across 7 of the 10 provinces of the country. In addition, 4.9% (*n* = 3) of the hospitals (one FLH and a TLH from Lusaka Province and one SLH from Western Province) were equipped with at least one zero degree endoscope while only 3.3% (*n* = 2) hospitals, both located in Lusaka Urban District, owned flexible rhinolaryngoscopes. It was further observed that 1.6% (*n* = 1) hospital had a fluoroscopy machine and 1.6% (*n* = 1) had an MRI scan. The study also revealed that 13.1% (*n* = 8) hospitals had computed tomography (CT) scanners and there was only 1.6% (*n* = 1) hospital with a radiotherapy equipment. No hospital was equipped with facial nerve monitors, videonystagmography (VNG) equipment, voice prosthesis for insertion post laryngectomy or PET (Positron Emission Tomography) scanning equipment.

## Discussion

ENT diseases continue to attract little attention despite being a major cause of morbidity and mortality worldwide [16, 17, 19]. Previous studies have consistently reported under-resourced, understaffed and outdated ENT, audiology and speech therapy services in the majority of low income countries [5, 16, 19, 23]. Zambia, like many low income countries, currently lacks ENT human resources, infrastructure and equipment [13, 19]. The Zambian healthcare service referral pathway offering ENT services consists of, in ascending order, health centres, clinics, FLH, SLH and TLH. Generally, patients follow this referral pathway to access appropriate ENT, audiology and speech therapy services. First Level Hospitals are designed to offer the most basic, TLH the most advanced and SLH intermediate services [24, 25]. Most of the hospitals in Zambia meant to offer significant ENT services (83.3% of TLH and 89.5% of SLH) participated in the study. Therefore, the 41.7% participation of FLH is likely to have little impact on the results of this study as they were not designed to have ENT surgeons, Audiologists and Speech Therapists.

By 2012, the country had listed 109 hospitals which included six TLH, 19 SLH and 84 FLH [19]. Although several more have been added subsequently, there is still an absence of basic ENT infrastructure and tools needed to reduce the morbidity and mortality associated with ENT infections. Lusaka, Copperbelt and Southern Provinces have the most hospitals. This may be associated with development and population growth in these provinces. Furthermore, these are more developed and historically the most economically vibrant provinces in the country. On the other hand, FBO owned hospitals were established in rural places to serve the populations living far from a comprehensive health service.

We have shown that Zambia has a critical shortage of ENT human resources. According to Fegan et al. (2009) [5], Zambia had two ENT surgeons, one Audiologist and no Speech Therapist who served 12 million individuals in 2009. This translated to 0.017 ENT surgeons, 0.008 Audiologists and 0 Speech Therapists per 100,000 population, respectively. By 2015, Mulwafu et al. (2017) [19] reported marginal improvement in these respective ratios to 0.045, 0.006 and 0.006 per 100,000 population. Since 2009, the projected population has increased by more than 4.5 million [26]. Nevertheless, HPCZ validated data indicates only two ENT Surgeons have been added giving a ratio of 0.0237 ENT surgeons per 100,000 population. One Audiologist serves the entire projected national population of 16,887,720 (Ratio 0.0059 Audiologists per 100,000 population). These ratios have declined from those noted in the last study because the population expansion over the past decade has not been matched with corresponding increase in ENT human resource. This may be partly because the country does not have specialist training programmes for ENT surgery, Audiology and Speech Therapy.

Three ENT surgeons, one Audiologist and one Speech Therapist reported by the study were not registered with HPCZ as of 24th January 2018. The three HPCZ unregistered ENT surgeons may be the explanation Mulwafu et al. (2017) reported seven ENT surgeons countrywide in their 2015 survey [19]. These surgeons may not be qualified enough for registration as specialists but continued to offer specialist services in areas where specialised ENT surgeons are deficient. This requires follow up by both HPCZ and MoH Zambia. On further enquiry of the HPCZ unregistered Audiologist and Speech Therapist, it was reported that these professionals were technicians offering limited services and inappropriately reported as Audiologist and Speech Therapist. The study further reported eight (21.1%) less General Surgeons than registered by the HPCZ. These were distributed across the 44.0% (*n* = 48) hospitals which did not participate in the study nationwide.

With almost all the core ENT and supporting health personnel based in Lusaka city, the rest of the country’s ENT service is minimal, and in many places, non-existent. As a result, hospitals offering ENT services are overwhelmed with patients as are many higher level hospitals in developing countries [25]. The large patient load inevitably leads to fewer medical specialists and nursing staff, with rapid depletion of the available resources. As a result, patient morbidity and mortality increases due to suboptimal handling of medical and surgical emergencies, poor nursing care, lack of critical care beds and surgical error emanating from health worker fatigue [27–29]. Lack of ENT human resource is not unique to Zambia. In 2013, the World Health Organisation published its multi-country assessment of national capacity to provide hearing care. It found that 64% of African countries had less than one ENT specialist per million population, 81% had less than one Audiologist per million population and 19% had less than one Speech Therapist per million population. With developed nations having up to 13.325 ENT surgeons per 100,000 population and 12.477 Audiologists per 100,000 population, the ENT human resource discrepancy between the high and low income countries need urgent correction [8, 30].

To adequately address the shortage of ENT human resource, The Zambian Ministry of Health should engage with the more developed countries and establish collaborative programmes aimed at continuously training ENT, audiology and speech health personnel and acquisition of equipment. Several such collaborative programmes have been instrumental in advancing ENT in Africa, exemplified by the CBM International assistance in the establishment of ENT units in Central Africa, The University of Cape Town Karl Storz Head and Neck Fellowship training African Head and Neck Fellows and Operation Ear Drop Kenya equipping the temporal bone laboratory in Nairobi [19].

ENT infrastructure exists at eight hospitals nationwide, mostly in the urban areas. All but one (FBO owned hospital) lacked modern and adequate ENT equipment, with some having consultation rooms as the only available ENT resources. These findings correspond with the details in the Zambia ENT National Strategic Plan 2017–2021 [31]. With health facilities in excess of 1956, of which about 88% are state owned, 13% private and 6% FBO owned [24], having nine ENT examination rooms, four audiology booths and one speech therapy room violates the WHO third sustainable development goal (SDG): The third SDG addresses good health and wellbeing for mankind. Even though there exists no agreed international definition of which specific services should be provided by the different levels of hospital care, WHO advises secondary, tertiary and national referral hospitals have Intensive Care Units (ICUs), ENT and speech therapy facilities. It also advises that audiology services should be available at tertiary and national referral hospitals to optimize hearing health [25]. Therefore, audiology services should be provided to all secondary hospitals in Zambia to achieve meaningful reduction in the prevalence of preventable hearing loss, especially that eight of the 10 provinces of Zambia (66.5% of the total country population) have secondary hospitals as their highest referral centres. The countrywide restriction to two hospitals of ABR, OAE and ASSR equipment suggests child hearing loss is not picked up early enough for effective intervention. This impacts on the patient’s quality of life, failing to develop proper speech and largely ending up in ‘schools for the deaf’.

Most hospitals lack ENT, audiological and speech therapy equipment. Ear syringing kits and pneumatic bulbs are basic ear equipment important in diagnosing and treating the most common ear conditions – occlusive ear wax and otitis media with effusion (OME). Lack of this equipment in many hospitals may increase the incidence of preventable hearing loss. With 90% of children having OME before school going age [32] and ear wax being the most common external ear canal obstructing pathology causing reversible hearing loss worldwide [33], these conditions remain occupational hazards for early childhood in Zambia. About a third of hospitals in Zambia own basic nasal instruments. This outcome may compromise diagnosis and management of epistaxis and other common nasal and sinus conditions.

Suction equipment is widely available among hospitals in Zambia because it is universally required in non-ENT patients like those under gynaecology and medicine. The lack of examination lights, however, makes the examination of the ear, nose and throat challenging and unreliable. The authors experience has been that many doctors use their mobile phone lighting for patient examination purposes. The lack of tongue depressors in over a third of the hospitals suggests hospitals may not appreciate the importance of stocking this relatively cheap tool.

The current study also indicates that specialised ENT, audiology and speech therapy equipment remain poorly stocked in Zambian hospitals. Most of the advanced ENT equipment was found at two hospitals in one city (Lusaka), which leaves this pair of hospitals overwhelmed with referrals from across the country.

The results from the current study showed that adenotonsillectomy and tracheostomy instrument sets are the most available advanced ENT equipment. Their wider availability is because tracheostomy, a lifesaving procedure, is largely performed by General Surgeons who are more readily available than ENT surgeons. Performance of tracheostomy and adenotonsillectomy by General Surgeons reduces the morbidity and mortality associated with upper airway obstruction. Adenotonsillectomy is less often performed by General Surgeons but is the most common procedure done by ENT surgeons on their operative state and private outreach programmes.

Across Zambia, only one hospital has a fluoroscopy machine for evaluation of swallow disorders and neck injuries, making these pathologies a diagnostic challenge. People needing fluoroscopy, as well as those requiring MRI scans have to travel to the one hospital in Lusaka to access the service, many of them having to traverse the country for many hours or days. Because these machines cater for a population in excess of 16.5 million, they frequently break down, which lengthens the waiting lists even further. Unfortunately, for cancer patients requiring prompt diagnoses and treatment, it is sometimes too late for effective treatment. Additionally, assessment of spread of malignancy to other areas of the body is made difficult by the absence of a PET scan in the country. These patients often end up with the oncology centre for palliation, where they also have to join the long treatment waiting list as there is only one radiotherapy centre in the country.

The challenges facing ENT services in low resourced countries is largely due to a lack of political will and shortage, maldistribution and inefficient use of resources [34]. Accordingly, WHO further noted the absolute need to boost political will to adequately finance health systems and efficiently use resources to achieve community universal access to health workers by 2030 [35]. The 48th World Health Assembly urged member states to prepare national plans to address hearing loss [34].

Despite the many challenges that have been highlighted in this paper, the government of Zambia has laid out strategies to reduce morbidity associated with ENT diseases by increasing infrastructure and equipment for ENT services, training ENT health personnel and strengthening existing personnel, educating the public on ENT conditions and creating an ENT society [13]. Following the rolling out of the National Health Strategic Plan 2017–2021, one tertiary hospital’s audiology department has recently been refurbished and received new equipment. Four doctors are currently in ENT specialist training.

### Study limitations

It was difficult to get all hospitals to respond. Like many developing world countries, Zambia’s communication network is poor. Many rural places do not have reliable internet, and postal services were often unreliable. We used the 2012 List of Health Facilities in Zambia in our survey. This document, released by the Ministry of Health of Zambia in 2013, was the most recent formal health list publication. This may have made the recorded absolute count of hospitals inaccurate because more health facilities have been established.

Owing to the shortage of ENT health personnel, some respondents to the questionnaire may not have been accurate in their reporting of procedures and equipment since they may not have been conversant with them. Where possible, images of equipment were confirmed with the authors by email and video conference calls.

## Conclusion

Zambia’s ENT services were poor at all levels of hospital care. There were absolute deficiencies in infrastructure, human resource and equipment in hospitals, critically needing intervention. Concerted efforts by both the Zambian government and collaborating partners are required to meaningfully improve the service. This data should be used to direct national policy on the improvement of ENT service provision in Zambia.

## Additional files

Additional file 1: Data collection tool. Hospital Respondents Questionnaire. Includes Respondents information, written consent and description of terms. (DOC 465 kb)

Additional file 2: Description of terms. Terms applicable to the Zambian health care system at the time the study was conducted. (DOCX 17 kb)

Additional file 3: STROBE checklist. Checklist used in writing the manuscript. (DOCX 18 kb)

## Data Availability

The data that has been used has been archived and stored at the University of KwaZulu-Natal. Data can be requested by following the guidelines laid in the Data Access Policy of the University of KwaZulu-Natal.

## Abbreviations

ABR: Auditory Brainstem Reflexes
ASSR: Auditory Steady State Response
CB: Copperbelt
Cent: Central
CT: Computed tomography
East: Eastern
ENT: Ear, Nose and Throat
FBO: Faith based organisation
FLH: First Level Hospitals
FNAC: Fine needle aspiration cytology
GBD: Global Burden of Disease
HPCZ: Health Professions Council of Zambia
HRO: Human Resource Officer
ICU: Intensive Care Unit
LSK: Lusaka
Lua: Luapula
MoH: Ministry of Health
MOIC: Medical Officer-in-Charge
MRI: Magnetic resonance imaging
Much: Muchinga
NHSP: National Health Strategic Plan
North: Northern
N-West: North-Western
OAE: Otoacoustic Emission
OME: Otitis media with effusion
PET: Positron Emission Tomography
PH: Public Hospitals
PrH: Private Hospitals
SDG: Sustainable Development Goal
SLH: Second Level Hospitals
South: Southern
TLH: Third Level Hospitals
VNG: Videonystagmography
West: Western
WHO: World Health Organisation
