# Supplementary material for: Africa’s challenged ENT services: highlighting challenges in Zambia
Source: BMC Health Serv Res. 2019 Jul 2;19:443. doi: 10.1186/s12913-019-4267-y (PMC6604437; doi:10.1186/s12913-019-4267-y)
Supplement: Supplementary file 1 — Data collection tool. Hospital Respondents Questionnaire. Includes Respondents information, written consent and description of terms. (DOC 465 kb) [file 12913_2019_4267_MOESM1_ESM.doc]

**Hospital Questionnaire Respondents Information**

Dear Health Worker,

My name is Dr Lufunda Lukama, a Medical Doctor at the University Teaching Hospital in Lusaka and Zambian Government sponsored postgraduate student at the University of KwaZulu-Natal (UKZN) in Durban.

I am conducting a survey of the ENT (Ear, Nose and Throat) Services in Zambia, a study which involves all the hospitals. The result of this survey will be used to establish the magnitude of the difficulties the ENT service delivery faces in our country and help guide future planning on how to improve the ENT health care to the Zambian people. The survey will also contribute to my Master of Medicine in ENT and Head and Neck Surgery qualification.

Permission to conduct the Survey has been granted by the Ministry of Health, Zambia and ethically reviewed and approved by UKZN Biomedical Research Ethics Committee (approval number: BE 463/16) and The University of Zambia Research Ethics Committee (approval number REF.NO.010-12-16).

I would like to kindly request you to complete the attached questionnaire and return it to the address used to deliver it to you at your earliest convenience. It takes approximately 20 to 30 minutes to complete. Your name and position at the hospital, as well as the name of the hospital will remain anonymous in the publication, which makes the risk of breach of your confidentiality negligible.

Your participation in this study is voluntary and does not come with financial or material gain. However, it would be vital in progressing our country’s ENT service and ultimately improving the general health of Zambians.

For any questions and clarifications, please contact me on the address indicated below.

Regards,

**Dr Lufunda Lukama**, MB ChB

ENT Registrar

University Teaching Hospital, Lusaka, Zambia

University of KwaZulu-Natal, Durban, South Africa

Mobile Phones: +260 978 215191 (Zambia)

+27 72 305 0675 (South Africa)

Email: [lufundal@yahoo.com](mailto:lufundal@yahoo.com)

Work Address:

University of KwaZulu-Natal College of Health Sciences

Nelson R Mandela School of Medicine Department of Otorhinolaryngology, Head and Neck Surgery 5^th^ Floor, Room 546, 719 Umbilo Road Durban 4001

South Africa Tel: +27 (0) 31 260 4292

**Consent**

I, ……………………………….. (Name of Participant) have been informed about the study ‘Survey of Ear, Nose and Throat Services in Zambia’ by Dr Lufunda Lukama.

I understand the purpose, importance and cautions of the study and my questions and concerns have been satisfactorily addressed. I am aware that my participation in this study is voluntary and that my identification will remain confidential.

I understand that I may contact the researcher at the address below if I have any concerns/questions/queries related to the study.

University of KwaZulu-Natal College of Health Sciences Nelson R Mandela School of Medicine Department of Otorhinolaryngology, Head and Neck Surgery 5 th Floor, Room 546 Durban 4001, South Africa Mobile Phones: +260 978 215191 (Zambia) +27 72 305 0675 (South Africa) Email: [lufundal@yahoo.com](mailto:lufundal@yahoo.com)

If I have any questions or concerns about my rights as a study participant, of if I am concerned about an aspect of the study or the researchers then I may contact:

UNIVERSITY OF ZAMBIA BIOMEDICAL RESEARCH ETHICS COMMITTEE Ridgeway Campus P.O.Bax 50110, Lusaka, ZAMBIA Tel: +260-1-256067 Telex: UNZALU ZA 44370 Fax: +260-1-250753 Email: [unzarec@unza.zm](mailto:unzarec@unza.zm)

**OR**

BIOMEDICAL RESEARCH ETHICS ADMINISTRATION Research Office,Westville Campus Govan Mbeki Building Private Bag X 54001 Durban, 4000 KwaZulu-Natal, SOUTH FRICA Tel: +27 31 260 4769 Fax: +27 31 260 4609 Email: BREC@ukzn.ac.za

Signature of Participant ………………………… Date …………………

Signature of Witness ……………………….... Date …………………

**Hospital Survey Questionnaire**

**Hospital Code: H21**

**Hospital Level of Care** – For Public Hospitals- (1st, 2^nd^ or 3^rd^ Level): ….

**Hospital Catchment Population: …………………**

**Hospital Status: [**tick as appropriate]

1. Public
2. Private
3. Faith Based Organisation Owned (Mission)…

**Respondent’s Position at Hospital: ………………………………….**

|  |
| --- |

1. **INFRASTRUCTURE**

**Do you currently have a functional …** (Tick Yes or No in or against the space provided for each option)

1. ENT dedicated examination room? **Yes**  **No**
2. Operating theatre? **Yes**  **No**
3. Operating theatre time dedicated to ENT procedures? **Yes**   **No**
4. Audiology Booth? **Yes**  **No**
5. A room or building dedicated to speech and/or language therapy?

**Yes**  **No**

1. Functional Intensive/ Critical Care Facility? **Yes No**
2. Functional High Dependency/High Care Facility? **Yes No**
3. **HUMAN RESOURCE**
4. **Core ENT Workforce**
5. **How many of the following Health Professions Council of Zambia**

**(HPCZ) registered professionals do you currently have at this**

**hospital?**

**(**Write down the number in or against the space provided for each option)

1. Specialist (Qualified) ENT Surgeons
2. Registrars and stationed in ENT
3. Non-Specialist and/or Non-Registrar Medical Doctors stationed in ENT
4. Medical Licentiates with further training in ENT
5. Clinical Officers formally trained in ENT
6. Audiologists (with a degree or higher qualification in Audiology)
7. Speech therapists (with a degree or higher qualification in Speech Therapy)
8. Nurses dedicated to ENT Service (e.g. stationed in the ENT consulting rooms, wards, theatres, etc.)
9. **What is the current total number of the following HPCZ**

**registered professionals at this hospital?**

(Write down the number in or against the space provided for each option)

1. Specialist Doctors, including ENT Surgeons
2. Registrars
3. Non-Specialist and/or Non-Registrar Medical Doctors
4. Medical Licentiates
5. Clinical Officers
6. Nurses
7. **Supportive Professionals**

**How many of the following HPCZ registered professionals do you currently have at your facility?**

(Write down the number in or against the space provided for each option)

1. Plastic and Reconstructive Surgeons
2. Neurosurgeons
3. Ophthalmologists (Doctors specialized in Ophthalmology)
4. Maxillofacial Surgeons
5. Dental Surgeons
6. Vascular Surgeons
7. Thoracic Surgeons
8. **EQUIPMENT**

**The following equipment relates to ENT. Please indicate how many of each item are functional at your hospital.**

(Write down the number in or against the space provided for each option)

1. **Basic Equipment (Clinic Equipment)**
2. **Ear Equipment**
3. Otoscope (Auriscope)
4. Pneumatic bulb for otoscope
5. Tuning forks
6. 256 Hz
7. 512 Hz
8. 1024 Hz
9. Barany Box
10. Ear syringing kit (commercial or locally assembled)
11. Ear hook
12. Jobson horne probe
13. **Nose and Sinus Equipment**
14. Nasal speculum
15. Nasal packing forceps
16. Biopsy forceps
17. **Head and Neck Equipment**
18. Head lamp or examination light reflecting mirror
19. Laryngeal mirrors
20. Tongue depressor or wooden spatula
21. Suction equipment
22. Fine Needle Aspiration Biopsy apparatus
23. **Specialised Equipment**
24. **ENT Equipment**
    1. **For the Ear**
25. Examination (Clinic) microscope
26. Operating microscope
27. Myringotomy and Grommet insertion instrument set
28. Mastoid drill kit
29. Tympanomastoidectomy instrument set
    1. **For the Nose and Paranasal Sinuses**
30. Rigid rhinoscope
31. Zero degree
32. Thirty degree
33. Forty-Five degree
34. Seventy degree
35. Flexible rhinolaryngoscope
36. Endoscopic Sinus Surgery Instrument Set
37. Frontal trephine set
38. Endoscopic microdebrider
39. Bipolar forceps/Ligature clips for Sphenopalatine artery cauterization/ligation
    1. **For Head and Neck**
40. Stroboscope
41. Tonsillectomy and Adenoidectomy set
42. Tracheostomy instrument set
43. Speech Valve insertion kit
44. Facial Nerve Monitor
45. **Audiological Equipment**
46. Otoscope (Auriscope)
47. Ear Syringing
48. Audiometer
49. Screening
50. Diagnostic (including bone conduction transducer)
51. Tympanometer
52. ABR (Auditory Brainstem Response) Equipment
53. Screening
54. Diagnostic
55. OAE (Otoacoustic Emission) Equipment
56. Screening
57. Diagnostic
58. Hearing Aid (HA)
59. Computer
60. Programmer (HI PRO) or equivalent
61. Hearing Aid Software
62. Hearing Aid Evaluations- impressions
63. Hearing Aid Fittings
64. Aural Rehabilitation
65. Auditory Steady State Response (ASSR) Equipment
66. Acoustic Reflex Testing Equipment
67. Electronystagmography (ENG) Equipment
68. Videonystagmography (VNG) Equipment
69. **Speech Therapy Equipment and Devices**
70. Fibreoptic laryngoscope for Evaluation of swallow
71. Devices
72. Voice Prosthesis
73. Speaking valve
74. Humidifiers
75. Electrolarynx
76. Trache brushes
77. Mirrors
78. BIBS
79. Pacifiers
80. Specialised feeding bottles and cups
81. Plastic Spoons for baby feeding
82. Augmentative and Alternative Communication (ACC) Devices
83. Printer and Laminating Machines

**C. Supportive Specialised Equipment**

1. Fluoroscopy Equipment
2. CAT (CT) Scanner
3. MRI Scanner
4. PET Scanning Equipment
5. Radiotherapy Machine
6. **SURGICAL PROCEDURES**

**Kindly indicate which of the following procedures your facility**

**performs:**

1. **Procedures and Operations**

**Which of the following surgical procedures and operations do you perform at your facility? Indicate Yes or No against each option:**

1. **For the Ear**
2. Extraction of foreign bodies from the ear
3. Myringotomy and grommet insertion
4. Tympanoplasty
5. Mastoidectomy
6. Fitting of hearing aids
7. **For the Nose and Sinuses**
8. Extraction of foreign bodies from the nose
9. Septoplasty
10. Rhinoplasty
11. Frontal Trephination
12. External Ethmoidectomy
13. Endoscopic Sinus Surgery
14. **For Head and Neck**
15. Fine Needle Aspiration Cytology
16. Tonsillectomy
17. Adenoidectomy
18. Tracheostomy
19. Maxillectomy
20. Laryngectomy
21. Parotidectomy
22. Excision of Head and Neck Cancers
23. Neck Dissection
24. Reconstruction of defects left by excision of Head and Neck Cancers
25. **Reasons for non-performance of the procedures and operations**

**For the procedures and operations not done at your hospital, indicate against each option the reason why using the number code below**:

**1** for non-availability of instruments and/or equipment

**2** for non-availability of competent person to do it

**3**  for both no equipment and a competent person to do it

**4** for non-availability of theatre

**5** for non-availability of Critical Care Facilities i.e. ICU

**6**  for both no theatre and no Critical Care Facilities

**7** for non-applicable

- 1. **For the Ear**
  2. Extraction of foreign bodies from the ear
  3. Myringotomy and grommet insertion
  4. Tympanoplasty
  5. Mastoidectomy
  6. Fitting of hearing aids
  7. **For the Nose and Sinuses**

1. Extraction of foreign bodies from the nose
2. Septoplasty
3. Rhinoplasty
4. Frontal Trephination
5. External Ethmoidectomy
6. Endoscopic Sinus Surgery
   1. **For Head and Neck**
   2. Fine Needle Aspiration Cytology
   3. Tonsillectomy
   4. Adenoidectomy
   5. Tracheostomy
   6. Maxillectomy
   7. Laryngectomy
   8. Parotidectomy
   9. Excision of Head and Neck Cancers
   10. Neck Dissection
   11. Reconstruction of defects left by excision of Head and Neck Cancers
7. **ESSENTIAL DRUGS**

**A) Do you stock the following medication?**

Indicate “**Yes”** or “**No”** against each option

1. **Medication used for Ear Diseases**
2. Amoxicillin or Amoxiclav
3. Erythromycin
4. Ciprofloxacin
5. Ceftriaxone
6. Acetic acid or other antiseptic ear drops
7. Quinolone ear drops e.g. ciprofloxacin or ofloxacin
8. Other antibiotic ear drops
9. **Medication used for Nasal Diseases**
10. Gauze coated with BIPP (Bismuth Iodoform Paraffin Paste) or other nasal packing antiseptic paste
11. Topical Nasal decongestants e.g. xylometazoline, oxymetazoline
12. Nasal steroid sprays/drops
13. Chlorpheniramine, cetirizine, loratidine or other antihistamine (oral or injectable)
14. Sodium Chromoglycate or other mast cell stabilizers nosedrops
15. Ipratropium Bromide or other anticholinergic intranasal spray
16. Montelukast or other Leukotriene Receptor Antagonist (oral formulation)
17. Prednisolone
18. Cloxacillin
19. Saline or bicarbonate nasal douche/wash
20. Systemic Antifungal agents e.g. Amphotericin B, Voriconazole
21. **Medication used for Throat Diseases**
22. Benzylpenicillin (X-Pen)
23. Penicillin V
24. Metronidazole (oral or injectable)
25. Clindamycin (oral or injectable)
26. Chloramphenicol (oral or injectable)
27. Injectable corticosteroids e.g. hydrocortisone, dexamethasone
28. Proton pump inhibitors e.g. omeprazole, lansoprazole (injectable or oral)
29. **For the medication you do not stock, what is the reason for not stocking it?**

Please indicate against each option with the code below:

**1** for not considered necessary

**2** for not affordable or expensive

**3** for both not necessary and not affordable

**4**  for no knowledge about the drug

**5**  for not applicable

1. **Medication used for Ear Disease**
2. Amoxicillin or Amoxiclav
3. Erythromycin
4. Ciprofloxacin
5. Ceftriaxone
6. Acetic acid or other antiseptic ear drops
7. Quinolone ear drops e.g. ciprofloxacin or ofloxacin
8. Other antibiotic ear drops
9. **Medication used for Nasal and Sinus Disease**
10. Gauze coated with BIPP (Bismuth Iodoform Paraffin Paste) or other nasal packing antiseptic paste
11. Topical Nasal decongestants e.g. xylometazoline, oxymetazoline
12. Nasal steroid sprays/drops
13. Chlorpheniramine, cetirizine, loratidine or other antihistamine (oral or injectable)
14. Sodium Chromoglycate or other mast cell stabilizers
15. Ipratropium Bromide or other anticholinergic intranasal spray
16. Montelukast or other Leukotriene Receptor Antagonist
17. Prednisolone
18. Cloxacillin
19. Saline or bicarbonate nasal douche/wash
20. **Medication used for Throat Disease**
21. Benzylpenicillin (X-Pen)
22. Penicillin V
23. Metronidazole (oral or injectable)
24. Clindamycin (oral or injectable)
25. Chloramphenicol (oral or injectable)
26. Injectable corticosteroids e.g. hydrocortisone, dexamethasone
27. **BUDGET AND ENT RELEVANCE PERCEPTION**

Indicate “**Yes”,** “**No” or “Not Sure”** against each option

1. Do you have a hospital budget specific for ENT?
2. If Yes, what proportion of the whole hospital budget is the ENT budget? Indicate as a percentage.
3. Is ENT an important branch of Clinical Practice?
4. Do you think the ENT service has received enough attention at your hospital?
5. Do you think ENT service delivery needs to be improved at your Hospital?

**You have come to the end of the Questionnaire. Thank you for your time.**

**END OF QUESTIONNAIRE**

**Description of Terms**

The terms described below are applicable to Zambia.

1. **Third Level Hospitals (**Specialist or Tertiary Hospitals)

The highest referral hospitals in Zambia, catering for a catchment population of approximately 800,000 and above, and have sub-specialisations in Internal Medicine, Surgery, Paediatrics, Obstetrics and Gynaecology, Intensive Care, Psychiatry, Training and Research. All complicated cases not attended to at second level hospitals are referred to third level hospitals. (The 2012 List of Health Facilities in Zambia Preliminary Report. Zambia. 2013).

The sub-specialisations include Nephrology, Rheumatology, Cardiology, Neurology, Radiology, Dermatology, Oncology, Infectious Disease, Endocrinology, Otorhinolaryngology, Neurosurgery, Ophthalmology, Maxillofacial Surgery, Paediatric Surgery, Neonatology, Nutrition and Dietetics. They may not be found at every Third Level Hospital.

1. **Second Level Hospitals (Provincial or General Hospitals)**

They are intended to cater for a catchment area of between 200,000 and 800,000 people, with services in Internal Medicine, General Surgery, Paediatrics, Obstetrics and Gynaecology, Dentistry, Psychiatry and Intensive Care. They also act as referrals for the first level institutions, including the provision of technical back up and training. (The 2012 List of Health Facilities in Zambia Preliminary Report. Zambia. 2013)

1. **First level hospitals (District Hospitals)**

They are the third highest levels of care after the Second and Third Level referral hospitals, serving a population of between 80,000 and 200,000 and providing Medical, Surgical, Obstetric and Diagnostic services and all clinical support of Health Centre referrals. (The 2012 List of Health Facilities in Zambia Preliminary Report. Zambia. 2013)

1. **Registrar**

A medical doctor training to be a specialist and has successfully completed at least one year of the Master of Medicine training programme.

1. **Medical Licentiate**

A clinician holding an Advanced Diploma in General Medicine or Specialty of Medicine or a Bachelor of Science in Clinical Science.

1. **Clinical Officer**

A Clinician holding a Diploma in Clinical Medical Sciences or equivalent.

1. **Private Hospital**

Hospital not owned by the State

1. **Faith Based Organisation**

Religious Organisation, often referred to as a Mission
