# Supplementary material for: Africa’s challenged ENT services: highlighting challenges in Zambia
Source: BMC Health Serv Res. 2019 Jul 2;19:443. doi: 10.1186/s12913-019-4267-y (PMC6604437; doi:10.1186/s12913-019-4267-y)
Supplement: Supplementary file 2 — Description of terms. Terms applicable to the Zambian health care system at the time the study was conducted. (DOCX 17 kb) [file 12913_2019_4267_MOESM2_ESM.docx]

**Description of Terms**

The terms described below are applicable to Zambia.

1. Third Level Hospitals (Specialist or Tertiary Hospitals)

The highest referral hospitals in Zambia, catering for a catchment population of approximately 800,000 and above, and have sub-specialisations in Internal Medicine, Surgery, Paediatrics, Obstetrics and Gynaecology, Intensive Care, Psychiatry, Training and Research. All complicated cases not attended to at second level hospitals are referred to third level hospitals (1).

The sub-specialisations include Nephrology, Rheumatology, Cardiology, Neurology, Radiology, Dermatology, Oncology, Infectious Disease, Endocrinology, Otorhinolaryngology, Neurosurgery, Ophthalmology, Maxillofacial Surgery, Paediatric Surgery, Neonatology, Nutrition and Dietetics. They may not be found at every Third Level Hospital.

1. Second Level Hospitals (Provincial or General Hospitals)

They are intended to cater for a catchment area of between 200,000 and 800,000 people, with services in Internal Medicine, General Surgery, Paediatrics, Obstetrics and Gynaecology, Dentistry, Psychiatry and Intensive Care. They also act as referrals for the first level institutions, including the provision of technical back up and training (1).

1. First level hospitals (District Hospitals)

They are the third highest levels of care after the Second and Third Level referral hospitals, serving a population of between 80,000 and 200,000 and providing Medical, Surgical, Obstetric and Diagnostic services and all clinical support of Health Centre referrals(1).

1. Registrar

A medical doctor training to be a specialist and has successfully completed at least one year of the Master of Medicine training programme.

1. Medical Licentiate

A clinician holding an Advanced Diploma in General Medicine or Specialty of Medicine or a Bachelor of Science in Clinical Science.

1. Clinical Officer

A Clinician holding a Diploma in Clinical Medical Sciences or equivalent.

1. Private Hospital

Hospital not owned by the State

1. Faith Based Organisation

Religious Organisation, often referred to as a Mission

**Reference**

1. Report P. The 2012 List of Health Facilities in Zambia Preliminary Report. 2013;(15).
